# Importance of Pre-anesthetic Evaluation in Diagnosing Coexisting Asymptomatic Medical Conditions: A Report of Two Cases

**DOI:** 10.7759/cureus.46250

**Published:** 2023-09-30

**Authors:** Ahmed Alanzi, Samar Ghazzal, Sara Abduljawad, Ameera Ghuloom, Amir Fouad, Shahid Adeel

**Affiliations:** 1 Anesthesia and Critical Care, King Hamad University Hospital, Muharraq, BHR; 2 General Practice, Arabian Gulf University, Manama, BHR; 3 General Practice, Ministry of Health - Bahrain, Manama, BHR

**Keywords:** surgery, anesthetic risk, perioperative risk, preoperative preparation, preoperative assessment

## Abstract

The preoperative assessment of patients undergoing surgery, often conducted in pre-anesthesia clinics, plays an important role in ensuring patient safety and optimizing perioperative outcomes. This assessment aids in identifying underlying medical conditions that might otherwise remain asymptomatic until they manifest as complications during or after surgery. Through these two case reports, the importance of pre-anesthesia assessment is highlighted. The first case involves a 67-year-old male whose surgery for lymph node excision was planned. However, during the preoperative assessment, atrial fibrillation and pulmonary hypertension were identified, necessitating further intervention and treatment adjustments before surgery. In the second case, an eight-year-old child with a history of vomiting and abdominal pain planned for tonsillectomy was discovered to have congenital hypothyroidism through a vigilant preoperative evaluation. Timely intervention and consultation with an endocrinologist ensured a safe surgery without complications. These cases emphasize the role of preoperative cardiovascular assessment, the utility of electrocardiograms (ECGs), and the relevance of routine laboratory tests in reducing perioperative mortality. Hence, pre-anesthesia assessments are not mere routine steps; they are essential components of patient care that significantly impact perioperative results.

## Introduction

An integral part of the perioperative care of a surgical patient is the preoperative evaluation (to assess the patient’s ability to withstand the procedure). It usually starts with the surgeon at the time of history-taking and examination. Pre-anesthesia evaluation (PAE) or pre-anesthesia check (PAC) are common names for the procedure performed by anesthesiologists. Both surgical and nonsurgical procedures typically start with PAC before administering anesthesia [1]. A PAC eliminates the need for unnecessary investigations and consultations, in addition to decreasing perioperative morbidity and death. Other advantages that can result from it include a decrease in case cancellations and delays as well as a shortening of hospital stays [2]. There is little doubt that thorough preoperative screening, investigation, and treatment are necessary for patients with moderate to severe comorbid illnesses. The care of patients who have no or few co-morbidities, typically those in American Society of Anesthesiologists (ASA) classes I and II, and who visit for regular elective surgery is ambiguous. In this case series, we report two patients of different ages, an elderly man diagnosed with atrial fibrillation and a young child diagnosed with hypothyroidism, in a preoperative anesthesia clinic, which underscores the importance of pre-anesthesia evaluation in detecting comorbid conditions and thus reducing the mortality related to anesthesia.

## Case presentation

Case report one

**Figure 1:**
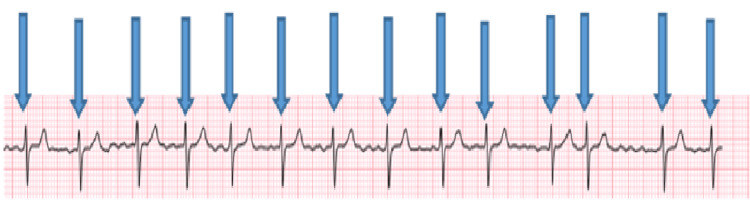
An electrocardiogram (ECG) showing characteristics of atrial fibrillation in the first case

We present the case of a 67-year-old male patient who had a history of smoking and alcohol abuse. His medical history revealed controlled hypothyroidism, and he was on levothyroxine treatment. Furthermore, the patient had a surgical history of eye surgery, endoscope, and wisdom tooth extraction under general anesthesia. He had no known allergies. As per hospital protocol, the patient was referred to the anesthesia clinic for an assessment regarding an elective excisional lymph node biopsy. An initial assessment of his vital signs showed a heart rate (HR) of 112, blood pressure (BP) of 118/70 mmHg, and respiratory rate (RR) of 20 breaths per minute. A physical examination showed an irregular pulse with no chest pain, dyspnea, or abnormal heart sounds. Chest and heart auscultation revealed clear breath sounds and normal heart sounds. Based on his history and initial assessment, the patient was classified as ASA class III. Further investigations revealed that the chest x-ray was unremarkable, and the ECG showed atrial fibrillation with premature ventricular contractions and a right axis deviation (Figure 1).

The patient's baseline laboratory results are shown in Table 1.

**Table 1:** Preoperative laboratory investigations include a complete blood count, coagulation profile, thyroid function test, electrolytes, liver function test, and glucose in the first case.

Indices	Level	Normal range
White blood cell count	8.62	4.5 to 11.0 × 10^9^/L
Hemoglobin level	14.7	13.8 to 17.2 g/dL
Platelet count	363	150 - 400 × 10^9^/L
Activated partial thromboplastin time (APTT)	23.6	28.8- 32.2 seconds
Prothrombin time (PT)	11	10.5-13.5 seconds
International normalized ratio (INR)	0.9	0.9- 1.12
Thyroid-stimulating hormone (TSH)	0.5	0.35-5.5 mIU/L
Triiodothyronine (T3) level	3.92	1.9-6.5 pmol/L
Thyroxine (T4) level	2.16	0.47-1.99 nmol/L
Sodium (Na) level	134	137-148 mmol/L
Potassium (K) level	5.2	3.5-5.1 mmol/L
Glucose level	6	3.9-5.6 mmol/L
Total bilirubin level	20.1	0-5 μmol/L
Direct bilirubin level	6.8	0-5 μmol/L
Alkaline phosphatase (ALP)	54.7	30- 130 U/L
Alanine transaminase (ALT) level	26.6	16-63 U/L
Gamma-glutamyl transferase (GGT)	47.7	16-63 U/L
Aspartate transaminase (AST)	21	16-63 U/L
Total protein level	70.6	64-82 g/L
Albumin level	47.2	38-50 g/L
Globulin level	23.4	38-50 g/L

Despite the absence of cardiac and respiratory symptoms and good effort tolerance, the patient was immediately counseled about the severity of the condition, and the decision was made to postpone the patient’s surgery until further investigations could be conducted. Consequently, an urgent cardiology referral was requested for further evaluation and management. The patient underwent a transesophageal endoscopy, which revealed a normal caliber of the main pulmonary artery and proximal branches. The estimated systolic pulmonary artery pressure was 50 mmHg, and the left ventricular cavity size was normal with a global systolic function of 55%. A 24-hour Holter monitoring was performed, revealing a maximum heart rate of 118 bpm and a minimum heart rate of 83 bpm. Normal sinus rhythm was observed with occasional ventricular premature complexes (VPCs). A diagnosis of atrial fibrillation was confirmed by the cardiologist. The patient underwent three unsuccessful attempts at cardioversion and was subsequently offered medical treatment and the CHA2DS2-VASC (congestive heart failure, hypertension, age, diabetes mellitus, stroke, vascular disease, age, sex) scoring system, which yielded a score of one. Therefore, anticoagulant therapy was not required at the time. Based on the previous investigations, he was labeled as a high-risk patient to proceed with surgery. Consent was obtained, explaining the potential risks and complications of both the surgical and anesthesia procedures, including the risk of arrhythmias and their consequences, such as stroke, end-organ damage, cardiac arrest, and the development of unstable atrial fibrillation intraoperatively, which may require cardioversion. The procedure was done under general anesthesia using propofol 1.5 mg/kg and fentanyl 1.5 mg/kg. A muscle relaxant was not given, as it was not needed in this case. The airway was secured using a laryngeal mask airway size four, and the maintenance of anesthesia was done using sevoflurane at 1%. Prophylactic cefazolin (2 g) was given before the incision started, as well as ondansetron (4 mg), dexamethasone (8 mg), paracetamol (1 mg IV), and diclofenac (75 mg IV). The procedure lasted for 45 minutes and was uneventful. The patient was taken to the recovery unit safely, monitored there for 45 minutes, and then shifted to the ward without any complications.

Case report two 

An eight-year-old child with no known case of any medical illness presented to the pre-anesthesia clinic for assessment as she was planned for tonsillectomy by the ENT team. On history, the father mentioned that she was born via C-section at 33 weeks with a low birth weight (1.9 kg), was a surviving twin, was admitted to the NICU for one day because of neonatal jaundice, and was discharged later. She was completely vaccinated and achieved all normal milestones except late tooth development. Systemic history was negative for fever, constipation, weight gain, cold intolerance, sleep disturbance, and hair loss. She has no family history of thyroid disease. Physical examination revealed clear exophthalmos, no periorbital puffiness, no goiter, and no lymphadenopathy. Her father mentioned that the child went to the emergency department one month before the presentation to the pre-anesthesia clinic due to three episodes of vomiting, non-bloody, non-bilious, non-projectile, and periumbilical abdominal pain with no radiation. It was associated with fatigue and decreased appetite. Initial lab work revealed extremely high thyroid-stimulating hormone (TSH) (>750 lU/mL), low free triiodothyronine (T3) and thyroxine (T4), anemia, normal WBC, and platelets (Table 2).

**Table 2:** Preoperative laboratory investigations include a complete blood count, thyroid function test, electrolytes, and renal function test in the second case.

Test	Result	Reference
White blood cells	7.9	4-14 x10^9/l
Hemoglobin level	10.2	11-15.5 g/dl
Red cell distribution width (RDW)	16.7	11.5:16 %
Platelet	195	150-450 x10^9/l
Thyroid-stimulating hormone (TSH)	>750.000	0.35-5.5 ulu/ml
Free triiodothyronine (T3) level	1.05	1.9-6.5 pg/ml
Free thyroxine (T4) level	0.19	0.47-1.99 ng/dl
Urea	6	3-7 mmol/l
Creatinine	54.5	27-62 umol/l
C-reactive protein (CRP) (mg/l)	<4.00	0.0-10 mg/l
Sodium (mmol/l)	135	137-148 mmol/l
Potassium (mmol/l)	3.7	3.5-5.1 mmol/l

**Figure 2:**
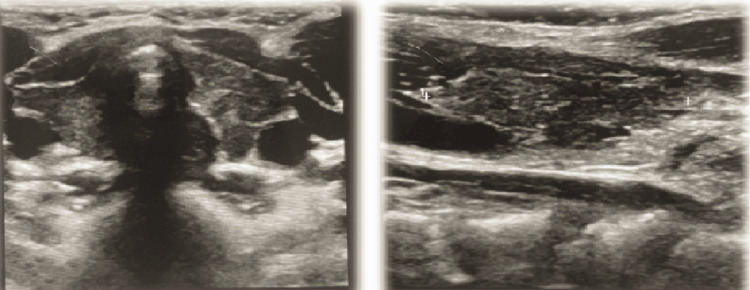
The neck ultrasound shows a mild form of chronic parenchymal changes of the thyroid gland in the second case.

The child was then referred to the pediatric endocrinologist for further assessment, evaluation, and management of the case. A thyroid ultrasound was ordered and showed a mild form of chronic parenchymal changes with slightly increased vascularity (Figure 2).

She was started on levothyroxine by the endocrinologist. Besides, she was considered safe to proceed with surgery under strict monitoring. The procedure lasted for 33 minutes and was uneventful. The patient was taken to the recovery unit safely, monitored there for 25 minutes, and then shifted to the ward without any complications.

## Discussion

Surgical procedures and the administration of anesthesia are associated with a complex stress response that is proportional to the magnitude of the injury, total operating time, amount of intraoperative blood loss, and degree of postoperative pain [3,4]. Preoperative medical evaluation's main objectives are to lower the patient's risk of surgical and anesthetic perioperative morbidity or mortality and to get him back to his ideal state of functioning as soon as possible [5]. While surgeons delve into the specifics of the intended procedure, anesthesiologists contribute significantly by assessing a patient’s overall health, identifying potential risks, and optimizing their readiness for surgery. This phase, often conducted in the pre-anesthesia clinic, provides a unique opportunity to uncover hidden medical conditions that might otherwise remain asymptomatic until they manifest as complications during or after surgery. The goal of this case series was to investigate how well pre-anesthesia assessment clinics (PACs) work to enhance the standards and safety of postoperative patient care. Over the past 50 years, perioperative mortality, including anesthesia-related mortality, has declined, which is significant in developed countries [6, 7], mainly because of new anesthetics, improved monitoring equipment and training, the availability of recovery rooms, and improved airway management [8]. However, a previous review found higher rates of morbidity and mortality in non-operating room anesthesia, which was attributed to limited preoperative evaluation [9,10]. An Australian study found that the death rate was six times higher in patients who had inadequate preoperative evaluations than in individuals who underwent comprehensive evaluations [11-13].

According to the case notes of the first patient, preoperative cardiovascular testing is also essential for the evaluation and accurate risk stratification of patients undergoing non-cardiac surgery. The patient’s history of arrhythmias, cardiac dysfunction, and pulmonary hypertension remained concealed until the pre-operative assessment. The 2007 guidelines of the American College of Cardiology and the American Heart Association (ACA/AHA) are currently used to preoperatively assess surgical patients [14]. The guidelines address perioperative risk assessment by evaluating potential ischemia, which is categorized into risk categories ranging from low to high, using a cardiac stress test [14]. In general, conditions like hypertension, congestive heart failure, cardiomyopathy, valvular disease, arrhythmias, conduction defects, pacemakers, and pulmonary vascular disease are linked to higher morbidity and mortality in surgical patients, and their presence should direct more in-depth evaluation [14]. Prior to surgery, the predictive utility of a standard ECG had been called into question. According to Liu et al., 75% of preoperative ECGs from a sample of 513 elderly patients who were undergoing various surgical operations showed abnormalities [15]. Recent research by Noordzij et al. assessed the additional usefulness of preoperative ECG abnormalities in the prediction of postoperative cardiovascular death [16]. Similarly, in our patient, the ECG revealed an irregular rhythm, leading to the diagnosis of Afib and reducing the risk of peri-operative anesthesia complications. Therefore, it is unreasonable to question the value of an ECG screening before surgery in asymptomatic patients having a range of surgical procedures. Apart from cardiac evaluation, routine laboratory tests are also important for pre-operative evaluation and anesthesia fitness. It was believed that routine laboratory testing wasn't helpful or economical for individuals who appeared healthy based on their clinical assessment and medical history. A clinician should consider the risk-benefit ratio of any lab test ordered. When studying a healthy population, 5% of patients will have results that fall outside the normal range. The results of the history and physical examination, the patient's age, and the difficulty of the surgery should all be taken into account when ordering lab tests [17]. Our patient’s history of delayed tooth eruptions gave us the hint of also doing thyroid function tests, as it is evident that the most common manifestation of congenital hypothyroidism is delayed eruption of tooth dentitions. Thus, through a vigilant preoperative assessment, the medical team identified hypothyroidism in a young child that could have posed significant challenges during and after surgery. Early intervention and collaboration with an endocrinologist exemplify how preoperative evaluations serve not only as diagnostic tools but also as gateways to multidisciplinary care. We believe that the cases discussed in this series underscore the importance of pre-anesthesia clinic evaluations in diagnosing coexisting asymptomatic medical conditions, exemplify the crucial role this assessment plays in reducing anesthesia complications, and add valuable insights to the existing literature on improving pre-anesthesia clinical assessment.

## Conclusions

In conclusion, the importance of preoperative anesthesia clinic evaluation in diagnosing coexisting asymptomatic medical conditions cannot be overstated. Beyond surgical considerations, this evaluation serves as an opportunity for early detection of underlying medical conditions, thereby contributing to the overall well-being of the patient. Through comprehensive medical histories, physical examinations, and medication reviews, anesthesia providers can tailor their approach to the patient’s individual needs, mitigating potential risks, including perioperative mortality, and optimizing outcomes. The cases discussed in this series highlight the critical importance of preoperative anesthesia clinic evaluations in diagnosing coexisting asymptomatic medical conditions and underscore the fact that a comprehensive pre-anesthesia assessment is not just a routine step before surgery; it is a vital component of patient care that can have a significant impact on perioperative outcomes.
